# Zinc-mediated efficient and selective reductive aza-Claisen rearrangement of *N*-allyl- and *N*-propargylaminoanthraquinones in ionic liquid

**DOI:** 10.55730/1300-0527.3557

**Published:** 2023-03-07

**Authors:** Fereshteh ALBOOYEH, Ghasem AGHAPOUR

**Affiliations:** School of Chemistry, Damghan University, Damghan, Iran

**Keywords:** *N*-Allylaminoanthraquinone, *N*-propargylaminoanthraquinone, zinc powder, reductive aza-Claisen rearrangement, ionic liquid

## Abstract

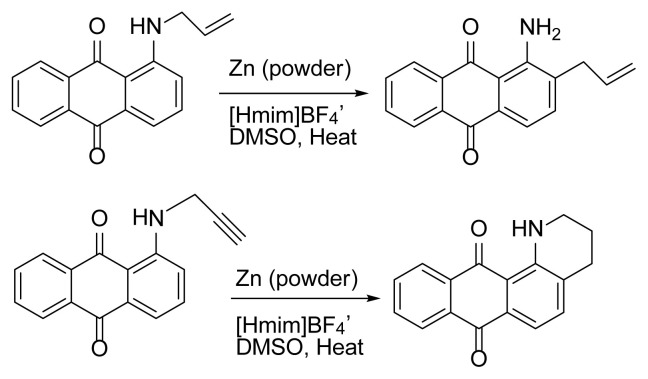

An efficient method is described for the first time for reductive aza-Claisen rearrangement of 1-(*N*-allylamino)anthraquinones to 1-amino-2-(prop-2′-enyl)anthraquinones using zinc powder in 1-methylimidazolium tetrafluoroborate ([Hmim] BF_4_) as an ionic liquid in good to excellent yields. Extending of this method on 1-(*N*-propargylamino)anthraquinones causes the production of 1,2,3,4-tetrahydronaphtho[2,3-*h*]quinoline-7,12-diones containing a newly synthesized six membered heterocyclic ring on anthraquinone core via performing this reductive [3,3] sigmatropic reaction followed by cyclization in a tandem manner. These rearrangements can be executed even in the presence of some other functional groups with excellent chemoselectivity.

## 1. Introduction

**Figure 1:**
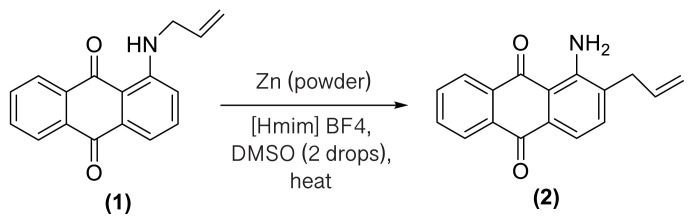
Reductive aza-Claisen rearrangement of 1-(*N*-allylamino)anthraquinone (**1**) to 1-amino-2-(prop-2′-enyl)anthraquinone (**2**) using zinc powder in ionic liquid.

Anthraquinone derivatives form a very important group in organic chemistry. These compounds have useful and important applications in different fields such as textile dyestuffs [1], colorimetric sensor systems [2,3], and pulp industry [4] and contain a lot of interesting and desired properties such as antioxidant [5], antifungal [6], enzyme inhibitor, and anti-Alzheimer [7], antiarthritic [8], antimalarial [9], hepatoprotective, neuroprotective, antidiabetes, and antiulcer [10], antimicrobial and antiviral [11], anticancer [12–16], laxative [17], and antiinflammatory [18]. Despite this importance, there are few ways for functionalization of anthraquinones. This is probably related to relative inertness of anthraquinone nucleus to perform electrophilic substitution reactions. Fortunately, the Claisen rearrangement [19] can be a solution for this matter and derivation of these valuable compounds. Despite different reports about this rearrangement on prop-2′-enyl(allyl)oxyanthraquinone derivatives [20, 21], as far as we know, there is no report about the aza-Claisen rearrangement [22–25] on anthraquinone systems. It must be noted that aza-Claisen rearrangement is generally less facile than its oxy-version, requires harsher reaction conditions, and affords lower yields of rearranged products [19, 25]. Now in the present work in continuation of our previous studies about this rearrangement [26–28] and also due to the importance of ionic liquids as environmentally friendly solvents in organic synthesis, we describe for the first time an efficient and selective method for the reductive aza-Claisen rearrangement of 1-(*N*-allylamino)anthraquinones to 1-amino-2-(prop-2′-enyl)anthraquinones using zinc powder in 1-methylimidazolium tetrafluoroborate ([Hmim] BF_4_) [29] as an ionic liquid and a small amount of DMSO (Figure 1).

## 2. Experimental

Solvents, reagents, and chemicals were obtained from Merck, Fluka, or Aldrich chemical companies. Substrates and products were characterized by their physical and spectral data. Fourier transform infrared (FTIR) spectra were recorded on a Perkin-Elmer RXI spectrophotometer. Nuclear magnetic resonance (NMR) spectra were recorded on Brucker Avance 300 and 400 spectrometers. Also, elemental analyses were performed using an Elementar vario EL III analyzer. Melting points were determined in open capillary tubes in an Electrothermal 9100 melting point apparatus. Thin layer chromatography (TLC) was carried out on silica gel 254 analytical sheets obtained from Fluka.

### 2.1. Typical procedure for the conversion of 1-aminoanthraquinone (3) to 1-(N-prop-2′-enylamino)anthraquinone (1)

Sodium hydroxide (1 mmol, 0.04 g) was added to a flask containing a solution of **3** (1 mmol, 0.22 g) in DMF (5 mL). The reaction mixture was stirred at 75 °C for 20 min so that its color changed from red to black. Next, allyl bromide (3 mmol, 0.26 mL) was added and stirring was continued for 6 h. Sodium hydroxide (0.5 mmol, 0.02 g) and then (after 15 min) allyl bromide (1 mmol, 0.09 mL) were added again and stirring was continued until TLC showed the completion of the reaction (30 h). The reaction mixture was cooled to room temperature and poured into distilled water (30 mL). The obtained precipitate was filtered and washed with distilled water (2 × 15 mL). **1** was obtained after column chromatography of this precipitate using a mixture of *n*-hexane and ethyl acetate (20:1) in 90% yield (0.24 g).

Similarly, 1-(*N*-prop-2′-ynylamino)anthraquinones were also synthesized from the reaction of 1-aminoanthraquinones with propargyl bromide via the above procedure.

### 2.2. Typical procedure for the reductive aza-Claisen rearrangement of 1-(*N*-prop-2′-enylamino)anthraquinone (1) to 1-amino-2-(prop-2′-enyl)anthraquinone (2)

Zinc powder (2 mmol, 0.13 g) was added to a flask containing a stirring solution of **1** (1 mmol, 0.26 g) in a mixture of [Hmim] BF_4_ (0.25 g) and DMSO (2 drops) in an oil bath at 160 °C. The progress of the reaction was monitored by TLC. After completion of the reaction (1 h), the reaction mixture was cooled to room temperature. Dichloromethane (2 × 20 mL) was added to it and then filtered. The organic layer was washed with distilled water (2 × 30 mL), saturated NaHCO_3_ (40 mL), and finally brine (40 mL) and evaporated after drying on calcium chloride. **2** was obtained after column chromatography of crude mixture on silica gel 60 using petroleum ether: ethyl acetate (40:1) as eluent in 90% yield (0.24 g) together with **3** in 6% yield (0.013 g).

Similarly, the reductive aza-Claisen rearrangement of 1-(*N*-prop-2′-ynylamino)anthraquinones were also carried out via this procedure except that the reaction temperature dropped to 140 °C.

### 2.3. Data

#### 2.3.1. *N*-Allyl- and *N*-propargylaminoanthraquinones

##### 1-(*N*-Prop-2′-enylamino)anthraquinone (1)

Dark red powder; mp = 122–124 °C; ^1^H NMR (CDCl_3_, 300 MHz): δ 3.96–4.00 (m, 2H), 5.23–5.26 (dd, 1H, *J* = 10.3, 1.2 Hz), 5.31–5.37 (dd, 1H, *J* = 17.2, 1.2 Hz), 5.92–6.05 (m, 1H), 6.98–7.01 (d, 1H, *J* = 8.4 Hz), 7.50–7.57 (m, 2H), 7.68–7.75 (m, 2H), 8.20–8.26 (m, 2H), 9.84 (s, br, 1H) ppm; ^13^C NMR (CDCl_3_, 75 MHz): δ 45.1, 113.1, 115.8, 116.6, 118.1, 126.6, 132.91, 132.97, 133.6, 133.7, 133.8, 134.5, 134.9, 135.1, 151.5, 183.6, 185.0 ppm; IR (KBr): 3422 (br), 3070 (w), 2924 (m), 2852 (w), 1651 (m), 1633 (m), 1628 (s), 1595 (s), 1303 (s), 1272 (s), 1071 (s), 802 (w), 735 (w), 708 (m) cm^−1^; Anal. Calcd for C_17_H_13_O_2_N: C, 77.56; H, 4.94; N, 5.32. Found: C, 77.01; H, 4.92; N, 4.96.

##### 1-Amino-5-(*N*- prop-2′-enylamino)anthraquinone (4)

Pale red crystals; mp = 158 °C; ^1^H NMR (CDCl_3_, 300 MHz): δ 3.98 (s, 2H), 5.22–5.25 (d, 1H, *J* = 10.1 Hz), 5.31–5.36 (d, 1H, *J* = 17.1 Hz), 5.94–6.03 (m, 1H), 6.45–6.95 (m, 4H),7.39–7.61 (m, 4H), 9.83 (br, 1H) ppm; ^13^C NMR (CDCl_3_, 75 MHz): δ 45.1, 113.3, 113.6, 115.1, 116.4, 116.5, 116.9, 121.6, 133.7, 134.5, 135.0, 135.9, 139.2, 150.6, 151.2, 185.4, 185.6 ppm; IR (KBr): 3415 (br), 3285 (br), 3012 (w), 2955 (w), 2865 (w), 1671 (s), 1650 (s), 1607 (s), 1533 (s), 1407 (s), 1366 (w), 1253 (s), 1090 (w), 893 (w), 793 (w), 722 (w) cm^−1^; Anal. Calcd for C_17_H_14_O_2_N_2_: C, 73.38; H, 5.03; N, 10.07. Found: C, 73.28; H, 5.01; N, 10.01.

##### 1,5-Bis(*N*- prop-2′-enylamino)anthraquinone (6)

Dark red powder; mp = 163–165 °C; ^1^H NMR (CDCl_3_, 300 MHz): δ 3.98 (s, br, 4H), 5.21–5.25 (d, 2H, *J* = 10.1 Hz), 5.30–5.36 (d, 2H, *J* = 17.2 Hz), 5.92–6.03 (m, 2H), 6.91–6.93 (d, 2H, *J* = 8.0 Hz), 7.46–7.57 (m, 4H), 9.82 (br, 2H) ppm; ^13^C NMR (CDCl_3_, 75 MHz): δ 45.6, 115.4, 116.9, 117.1, 127.1, 134.2, 135.4, 136.5, 151.6, 185.9 ppm; IR (KBr): 3358 (br), 3083 (w), 3052 (w), 2957 (w), 2867 (w), 1648 (s), 1538 (m), 1401 (m), 1366 (w), 1251 (s), 1009 (w), 893 (w), 796 (w), 721 (w) cm^−1^; Anal. Calcd for C_20_H_18_O_2_N_2_: C, 75.47; H, 5.66; N, 8.80. Found: C, 75.11; H, 5.96; N, 7.97.

##### 1-Amino-4-(*N*-prop-2′-enylamino)anthraquinone (9)

Violet powder; mp = 132–134 °C; ^1^H NMR (CDCl_3_, 300 MHz): δ 4.00–4.03 (s, br, 2H), 5.21–5.24 (d, 1H, *J* = 10.2 Hz), 5.28–5.33 (d, 1H, *J* = 17.1 Hz), 5.91–6.04 (m, 1H), 6.91–7.11 (m, 4H), 7.66–7.70 (m, 2H), 8.30–8.34 (m, 2H), 10.72 (s, br, 1H) ppm; ^13^C NMR (CDCl_3_, 75 MHz): δ 45.0, 109.8, 110.7, 116.5, 122.8, 126.13, 126.19, 128.1, 128.5, 132.1, 132.4, 134.2, 134.7, 144.4, 146.8, 182.7, 183.6 ppm; IR (KBr): 3375 (br), 3252 (br), 3069 (w), 2923 (s), 2853 (s), 1687 (m), 1650 (m), 1614 (s), 1597 (s), 1572 (s), 1535 (s), 1265 (s), 1169 (s), 813 (m), 794 (m), 725 (s) cm^−1^; Anal. Calcd for C_17_H_14_O_2_N_2_: C, 73.38; H, 5.03; N, 10.07. Found: C, 73.18; H, 4.99; N, 9.98.

##### 1,4-Bis(*N*-prop-2′-enylamino)anthraquinone (11)

Blue powder; mp = 140 °C; ^1^H NMR (CDCl_3_, 300 MHz): δ 4.04–4.07 (m, 4H), 5.21–5.35 (m, 4H), 5.95–6.04 (m, 2H), 7.16 (s, 2H), 7.69–7.72 (m, 2H), 8.33–8.36 (m, 2H), 10.82 (s, br, 2H) ppm; ^13^C NMR (CDCl_3_, 75 MHz): δ 45.4, 110.6, 116.9, 124.0, 126.4, 127.1, 132.5, 134.7, 146.4, 183.2 ppm; IR (KBr): 3426 (br), 3073 (w), 3011 (w), 2919 (m), 2850 (m), 1639 (m), 1608 (s), 1592 (s), 1574 (s), 1554 (s), 1518 (s), 1275 (s), 1234 (s), 1170 (s), 1017 (s), 995 (s), 915 (m), 801 (m), 734 (s) cm^−1^; Anal. Calcd for C_20_H_18_O_2_N_2_: C, 75.47; H, 5.66; N, 8.80. Found: C, 75.47; H, 5.66; N, 8.80.

##### 1-(*N*-Methyl-*N*-prop-2′-enylamino)anthraquinone (13)

Dark red powder; mp = 155–157 °C; ^1^H NMR (CDCl_3_, 300 MHz): δ 2.98 (s, 3H), 4.11–4.14 (m, 2H), 5.14–5.23 (m, 2H), 5.88–5.97 (m, 1H), 7.19–7.73 (m, 5H), 8.17–8.38 (m, 2H) ppm; ^13^C NMR (CDCl_3_, 75 MHz): δ 29.6, 42.9, 114.8, 116.7, 117.8, 126.7, 126.8, 131.6, 132.9, 133.1, 133.7, 133.83, 133.89, 134.8, 134.9, 149.3, 183.3, 184.9 ppm; IR (KBr): 3012 (w), 2933 (m), 2855 (w), 1689 (s), 1671 (m), 1663 (s), 1651 (s), 1505 (m), 1388 (m), 1290 (s), 930 (m), 858 (m) cm^−1^; Anal. Calcd for C_18_H_15_O_2_N: C, 77.97; H, 5.41; N, 5.05. Found: C, 78.00; H, 5.11; N, 5.00.

##### 1-(*N*-Methylamino)-5-(*N*′-prop-2′-enylamino)anthraquinone (15)

Red powder; mp = 187 °C; ^1^H NMR (CDCl_3_, 300 MHz): δ 2.96 (s, 3H), 4.31–4.34 (m, 2H), 5.14–5.23 (m, 2H), 5.86–6.00 (m, 1H), 7.30–7.73 (m, 5H), 8.17–8.23 (m, 1H), 8.71 (s, br, 1H), 8.84 (s, br, 1H) ppm; ^13^C NMR (CDCl_3_, 75 MHz): δ 26.1, 53.8, 111.3, 116.7, 117.8, 126.5, 126.7, 131.6, 132.9, 133.1, 133.7, 133.83, 133.89, 134.8, 134.9, 149.6, 181.3, 185.4 ppm; IR (KBr): 3427 (br), 3270 (br), 3076 (w), 2923 (s), 2852 (m), 1661 (w), 1647 (w), 1621 (s), 1600 (s), 1572 (m), 1514 (m), 1397 (m), 1263 (s), 1076 (m), 768 (m), 708 (m) cm^−1^; Anal. Calcd for C_18_H_16_O_2_N_2_: C, 73.97; H, 5.47; N, 9.58. Found: C, 73.87; H, 5.47; N, 9.07.

##### 1-(*N*-Prop-2′-ynylamino)anthraquinone (17)

Orange crystals; mp = 182–183 °C; ^1^H NMR (CDCl_3_, 300 MHz): δ 2.29–2.30 (t, 1H, *J* = 2.4 Hz), 4.14–4.17 (dd, 2H, *J* = 5.7, 2.4 Hz), 7.14–7.18 (dd, 1H, *J* = 8.3, 1.0 Hz), 7.53–7.82 (m, 4H), 8.19–8.29 (m, 2H), 9.81 (br, 1H) ppm; ^13^C NMR (CDCl_3_, 75 MHz): δ 32.4, 71.8, 79.4, 114.0, 116.5, 117.9, 126.7, 132.9, 133.1, 133.9, 134.6, 134.7, 135.3, 150.4, 183.5, 185.4 ppm; IR (KBr): 3441 (br), 3241 (s), 3075 (w), 2925 (m), 2853 (m), 2108 (w), 1671 (s), 1627 (s), 1592 (s), 1565 (s), 1507 (s), 1279 (s), 1229 (s), 1074 (s), 998 (s), 734 (s), 707 (s) cm^−1^; Anal. Calcd for C_17_H_11_O_2_N: C, 78.16; H, 4.21; N, 5.36. Found: C, 78.00; H, 4.38; N, 5.14.

##### 1-Amino-5-(*N*-prop-2′-ynylamino)anthraquinone (19)

Red powder; mp = 180–182 °C; ^1^H NMR (CDCl_3_, 300 MHz): δ 2.27–2.29 (t, 1H, *J* = 2.4 Hz), 4.14 (d, 2H, *J* = 2.1 Hz), 6.73 (br, 2H), 6.88–6.91 (dd, 1H, *J* = 8.3, 0.9 Hz), 7.08–7.11 (dd, 1H, *J* = 8.2, 1.1 Hz), 7.41–7.47 (m, 1H), 7.57–7.67 (m, 3H), 9.80 (br, 1H) ppm; ^13^C NMR (CDCl_3_, 75 MHz): δ 32.4, 71.7, 79.6, 115.8, 116.6, 116.7, 121.9, 128.7, 131.6, 134.6, 135.2, 135.7, 150.2, 150.6, 152.1, 185.4, 187.2 ppm; IR (KBr): 3420 (br), 3305 (br), 3070 (w), 2923 (s), 2852 (m), 2106 (w), 1665 (m), 1634 (s), 1602 (s), 1542 (s), 1503 (s), 1280 (s), 1166 (s), 1077 (s), 801 (m), 769 (m), 708 (s) cm^−1^; Anal. Calcd for C_17_H_12_O_2_N_2_: C, 73.91; H, 4.34; N, 10.14. Found: C, 73.64; H, 4.69; N, 10.11.

##### 1-Amino-4-(*N*-prop-2′-ynylamino)anthraquinone (21)

Violet powder; mp = 172 °C; ^1^H NMR (CDCl_3_, 300 MHz): δ 2.28 (s, 1H), 4.17 (s, 2H), 7.02–7.26 (m, 4H), 7.69–7.72 (m, 2H), 8.30–8.33 (m, 2H), 10.54 (br, 1H) ppm; ^13^C NMR (CDCl_3_, 75 MHz): δ 32.3, 71.8, 79.9, 111.0, 122.5, 126.2, 128.31, 128.37, 132.4, 132.5, 134.1, 134.5, 134.6, 144.6, 145.5, 183.5, 183.6 ppm; IR (KBr): 3411 (br), 3355 (br), 3283 (br), 3055 (w), 2956 (w), 2866 (w), 2126 (w), 1659 (s), 1651 (s), 1539 (m), 1401 (m), 1251 (s), 1007 (w), 891 (w), 796 (w), 723 (w) cm^−1^; Anal. Calcd for C_17_H_12_O_2_N_2_: C, 73.91; H, 4.34; N, 10.14. Found: C, 73.64; H, 4.68; N, 10.10.

#### 2.3.2. Products

##### 1-Amino-2-(prop-2′-enyl)anthraquinone (2)

Red powder; mp = 142–143 °C; ^1^H NMR (CDCl_3_, 300 MHz): δ 3.32–3.34 (d, 2H, *J* = 6.1 Hz), 5.14–5.23 (m, 2H), 5.88–5.97 (m, 1H), 7.06 (br, 2H), 7.30–7.32 (d, 1H, *J* = 7.5 Hz), 7.54–7.57 (d, 1H, *J* = 7.5 Hz), 7.64–7.73 (m, 2H), 8.17–8.24 (m, 2H) ppm; ^13^C NMR (CDCl_3_, 75 MHz): δ 36.1, 113.3, 116.8, 117.8, 126.5, 126.7, 131.6, 132.9, 133.1, 133.7, 133.81, 133.88, 134.8, 134.9, 149.6, 183.3, 185.4 ppm; IR (KBr): 3429 (s, br), 3295 (m, br), 3074 (w), 3007 (w), 2922 (w), 2852 (w), 1658 (s), 1634 (m), 1608 (s), 1591 (s), 1552 (s), 1425 (s), 1326 (s), 1279 (s), 747 (m), 712 (s) cm^−1^; Anal. Calcd for C_17_H_13_O_2_N: C, 77.56; H, 4.94; N, 5.32. Found: C, 76.90; H, 4.67; N, 5.11.

##### 1,5-Diamino-2-(prop-2′-enyl)anthraquinone (5)

Pale red powder; mp = 155 °C; ^1^H NMR (CDCl_3_, 300 MHz): δ 3.38–3.40 (d, 2H, *J* = 6.0 Hz), 5.17–5.26 (m, 2H), 5.98 (m, 1H), 6.81 (br, 2H), 6.88–6.91 (dd, 1H, *J* = 8.3, 1.0 Hz), 7.05 (br, 2H), 7.37–7.46 (m, 2H), 7.60–7.64 (m, 2H) ppm; ^13^C NMR (CDCl_3_, 75 MHz): δ 37.0, 113.3, 116.1, 116.6, 117.7, 121.7, 130.4, 134.0, 134.5, 135.0, 135.8, 140.7, 149.4, 150.6, 153.0, 185.6, 185.9 ppm; IR (KBr): 3431 (br), 3306 (br), 3074 (w), 2924 (s), 2852 (s), 1636 (m), 1598 (s), 1548 (s), 1459 (m), 1262 (s), 771 (w), 724 (w) cm^−1^; Anal. Calcd for C_17_H_14_O_2_N_2_: C, 73.38; H, 5.03; N, 10.07. Found: C, 73.14; H, 5.05; N, 10.07.

##### 1-Amino-2-(prop-2′-enyl)-5-(N-prop-2′-enylamino)anthraquinone (7)

Pink powder; mp = 154–156 °C; ^1^H NMR (CDCl_3_, 300 MHz): δ 3.38–3.40 (d, 2H, *J* = 6.1 Hz), 3.98–4.02 (m, 2H), 5.22–5.34 (m, 4H), 5.91–6.05 (m, 2H), 6.94–6.97 (dd, 1H, *J* = 8.4, 1.1 Hz), 7.07 (br, 2H), 7.37–7.39 (d, 1H, *J* = 7.6 Hz), 7.51–7.54 (m, 1H), 7.58–7.77 (m, 2H), 9.84 (br, 1H) ppm; ^13^C NMR (CDCl_3_, 75 MHz): δ 36.2, 45.2, 113.5, 115.3, 116.1, 116.6, 116.9, 117.7, 126.8, 128.7, 130.2, 130.8, 133.3, 133.8, 134.0, 135.1, 136.2, 149.4, 185.5, 186.0 ppm; IR (KBr): 3415 (br), 3285 (br), 3013 (w), 2955 (w), 2865 (w), 1671 (s), 1650 (s), 1607 (s), 1533 (s), 1407 (s), 1366 (w), 1253 (s), 1090 (w), 893 (w), 793 (w) cm^−1^; Anal. Calcd for C_20_H_18_O_2_N_2_: C, 75.47; H, 5.66; N, 8.80. Found: C, 75.27; H, 5.16; N, 8.80.

##### 1,5-Diamino-2,6-bis(prop-2′-enyl)anthraquinone (8)

Orange powder; mp = 161 °C; ^1^H NMR (CDCl_3_, 300 MHz): δ 3.39–3.41 (d, 4H, *J* = 6.0 Hz), 5.18–5.26 (m, 4H), 5.94–6.03 (m, 2H), 7.05 (br, 4H), 7.38–7.40 (d, 2H, *J* = 7.6 Hz), 7.62–7.65 (d, 2H, *J* = 7.6 Hz) ppm; ^13^C NMR (CDCl_3_, 75 MHz): δ 36.2, 114.0, 116.2, 116.9, 117.7, 130.8, 134.0, 135.1, 149.4, 185.5 ppm; IR (KBr): 3487 (br), 3322 (br), 3059 (w), 3025 (m), 2922 (s), 2851 (s), 1632 (m), 1592 (s), 1550 (s), 1492 (s), 1452 (s), 1275 (s), 915 (m), 758 (s), 698 (s) cm^−1^; Anal. Calcd for C_20_H_18_O_2_N_2_: C, 75.47; H, 5.66; N, 8.80. Found: C, 75.37; H, 5.56; N, 8.70.

##### 1,4-Diamino-2-(prop-2′-enyl)anthraquinone (10)

Purple powder; mp = 152–154 °C; ^1^H NMR (CDCl_3_, 400 MHz): δ 3.12–3.15 (d, 2H, *J* = 11.7 Hz), 4.46 (s, br, 2H), 4.84 (s, br, 2H), 4.87–4.89 (d, 1H, *J* = 6.7 Hz), 5.13–5.16 (d, 1H, *J* = 13.1 Hz), 5.89–6.00 (m, 1H), 7.20 (s, 1H), 7.84–7.85 (m, 2H), 8.28–8.29 (m, 2H) ppm; ^13^C NMR (CDCl_3_, 100 MHz): δ 35.9, 110.0, 113.1, 115.9, 120.0, 126.7, 126.8, 129.6, 132.1, 133.62, 133.63, 136.5, 136.6, 139.7, 140.0, 185.70, 185.74 ppm; IR (KBr): 3402 (br), 3274 (br), 3029 (w), 2963 (w), 2870 (w), 1650 (s), 1630 (s), 1610 (s), 1536 (s), 1455 (s), 1367 (w), 1258 (m), 898 (w), 799 (w) cm^−1^; Anal. Calcd for C_17_H_14_O_2_N_2_: C, 73.38; H, 5.03; N, 10.07. Found: C, 74.04; H, 4.59; N, 10.00.

##### 1-Amino-2-(prop-2v-enyl)-4-(*N*-prop-2′-enylamino)anthraquinone (12)

Blue powder; mp = 149–151 °C; ^1^H NMR (CDCl_3_, 400 MHz): δ 3.21 (m, 2H), 3.94 (m, 2H), 4.76 (br, 2H), 4.87 (m, 1H), 5.12–5.33 (m, 3H), 5.74 (br, 1H), 5.82–6.02 (m, 2H), 7.16 (s, 1H), 7.85 (m, 2H), 8.29 (m, 2H) ppm; ^13^C NMR (CDCl_3_, 100 MHz): δ 35.9, 46.1, 107.6, 113.3, 115.9, 117.44, 117.47, 126.87, 126.89, 129.3, 132.10, 132.12, 133.6, 135.5, 136.5, 138.0, 139.7, 185.77, 185.79 ppm; IR (KBr): 3410 (br), 3275 (br), 3041 (w), 2955 (w), 2865 (w), 1649 (s), 1628 (s), 1605 (s), 1536 (s), 1407 (s), 1253 (m), 799 (w), 726 (w) cm^−1^; Anal. Calcd for C_20_H_18_O_2_N_2_: C, 75.47; H, 5.66; N, 8.80. Found: C, 75.17; H, 5.46; N, 8.50.

##### 1-(*N*-Methylamino)-2-(prop-2′-enyl)anthraquinone (14)

Dark red powder; mp = 170 °C; ^1^H NMR (CDCl_3_, 300 MHz): δ 2.98 (s, 3H), 3.31–3.34 (m, 2H), 5.14–5.23 (m, 2H), 5.88–5.97 (m, 1H), 6.77 (br, 1H), 7.30–7.32 (d, 1H, *J* = 7.5 Hz), 7.54–7.57 (d, 1H, *J* = 7.5 Hz), 7.63–7.73 (m, 2H), 8.17–8.24 (m, 2H) ppm; ^13^C NMR (CDCl_3_, 75 MHz): δ 29.8, 36.9, 113.8, 116.7, 117.8, 126.7, 126.8, 131.6, 132.9, 133.1, 133.7, 133.83, 133.89, 134.8, 134.9, 149.6, 183.5, 185.8 ppm; IR (KBr): 3418 (br), 3073 (w), 2923 (s), 2852 (s), 1666 (s), 1625 (m), 1592 (m), 1518 (m), 1464 (m), 1258 (s), 714 (s) cm^−1^; Anal. Calcd for C_18_H_15_O_2_N: C, 77.97; H, 5.41; N, 5.05. Found: C, 77.77; H, 5.31; N, 5.04.

##### 1-Amino-5-(*N*-methylamino)-2-(prop-2′-enyl)anthraquinone (16)

Bright red powder; mp = 185–187 °C; ^1^H NMR (CDCl_3_, 300 MHz): δ 2.67 (s, 3H), 3.31–3.34 (m, 2H), 5.14–5.23 (m, 2H), 5.88–5.97 (m, 1H), 7.06 (br, 2H), 7.30–7.32 (d, 1H, *J* = 7.6 Hz), 7.54–7.57 (d, 1H, *J* = 7.5 Hz), 7.63–7.73 (m, 2H), 7.90–7.94 (m, 1H), 8.23 (br, 1H) ppm; ^13^C NMR (CDCl_3_, 75 MHz): δ 24.6, 36.1, 113.3, 116.7, 117.8, 126.5, 126.7, 131.6, 132.9, 133.1, 133.7, 133.83, 133.89, 134.8, 134.9, 149.8, 182.3, 185.4 ppm; IR (KBr): 3414 (br), 3283 (br), 3084 (w), 2956 (w), 2866 (w), 1656 (s), 1646 (s), 1534 (m), 1401 (m), 1254 (s), 1007 (w), 798 (w), 726 (w) cm^−1^; Anal. Calcd for C_18_H_16_O_2_N_2_: C, 73.97; H, 5.47; N, 9.58. Found: C, 73.77; H, 5.32; N_,_ 9.48.

##### 1,2,3,4-tetrahydronaphtho[2,3-*h*]quinoline-7,12-dione (18)

Orange powder; mp = 185–186 °C; ^1^H NMR (CDCl_3_, 300 MHz): δ 1.96–2.02 (m, 2H), 2.84–2.89 (m, 2H), 3.52–3.56 (m, 2H), 7.14–7.22 (m, 1H), 7.46–7.49 (d, 1H, *J* = 7.4 Hz), 7.61–7.77 (m, 2H), 8.21–8.28 (m, 2H), 9.81–9.94 (br, 1H) ppm; ^13^C NMR (CDCl_3_, 75 MHz): δ 20.1, 29.6, 41.0, 115.6, 116.5, 117.9, 126.5, 126.6, 130.2, 132.6, 133.1, 133.6, 133.7, 135.3, 149.2, 183.6, 184.6 ppm; IR (KBr): 3286 (br), 3010 (w), 2926 (s), 2852 (s), 1663 (s), 1624 (s), 1591 (s), 1519 (s), 1323 (s), 1296 (s), 1271 (s), 1005 (s), 974 (s), 750 (m), 734 (m), 715 (s) cm^−1^; Anal. Calcd for C_17_H_13_O_2_N: C, 77.56; H, 4.94; N, 5.32. Found: C, 77.51; H, 5.00; N, 5.31.

##### 8-Amino-1,2,3,4-tetrahydronaphtho[2,3-*h*]quinoline-7,12-dione (20)

Red powder; mp = 180–182 °C; ^1^H NMR (CDCl_3_, 300 MHz): δ 1.96–2.01 (m, 2H), 2.84–2.89 (m, 2H), 3.52–3.56 (m, 2H), 6.89 (br, 2H), 7.14–7.22 (m, 1H), 7.46–7.49 (d, 1H, *J* = 7.4 Hz), 7.67–7.74 (m, 2H), 8.23–8.26 (m, 1H), 9.81–9.94 (br, 1H) ppm; ^13^C NMR (CDCl_3_, 75 MHz): δ 28.3, 29.6, 41.3, 115.6, 116.5, 117.9, 126.5, 130.2, 132.6, 133.1, 133.6, 133.7, 135.3, 149.7, 152.6, 183.2, 184.1 ppm; IR (KBr): 3328 (br), 3150 (br), 3054 (w), 2912 (w), 2830 (w), 1665 (s), 1632 (m), 1601 (s), 1517 (s), 1501 (s), 1495 (m), 1385 (m), 1308 (m), 1245 (s), 1115 (s), 833 (s), 763 (w) cm^−1^; Anal. Calcd for C_17_H_14_O_2_N_2_: C, 73.38; H, 5.03; N, 10.07. Found: C, 73.38; H, 5.03; N, 10.07.

##### 6-Amino-1,2,3,4-tetrahydronaphtho[2,3-*h*]quinoline-7,12-dione (22)

Purple powder; mp = 176–177 °C; ^1^H NMR (CDCl_3_, 400 MHz): δ 1.96 (m, 2H), 2.79 (m, 2H), 3.04 (m, 2H), 4.47 (br, 2H), 6.99 (br, 1H), 7.10 (s, 1H), 7.85 (m, 2H), 8.29 (m, 2H) ppm; ^13^C NMR (CDCl_3_, 100 MHz): δ 22.2, 27.0, 41.8, 110.77, 110.78, 119.3, 125.6, 126.88, 126.89, 132.11, 132.12, 133.62, 133.63, 138.0, 139.4, 185.7, 185.8 ppm; IR (KBr): 3340 (br), 3160 (br), 3066 (w), 2915 (w), 2822 (w), 1667 (s), 1632 (m), 1607 (s), 1518 (s), 1505 (s), 1388 (m), 1310 (m), 1246 (s), 1117 (s), 835 (s) 765 (w) cm^−1^; Anal. Calcd for C_17_H_14_O_2_N_2_: C, 73.38; H, 5.03; N, 10.07. Found: C, 73.38; H, 5.03; N, 10.07.

## 3. Results and discussion

First, we tried to identify the optimized reaction conditions for this rearrangement via its operation on 1-(*N*-prop-2′-enylamino)anthraquinone **1** as a model compound. For this propose, zinc powder was used in different reaction conditions (Table 1).

As shown in Table 1, performing this reaction using zinc powder (2 equiv.) in solvents such as AcOH, DMF, and DMSO under heating up to 160 °C was unsuccessful so that the desired product **2** was formed in only 20%–30% yield with concomitant formation of the undesired deallylated product **3** in large amounts after 4.5–5 h (Table 1, entries 1–3). Surprisingly, the aza-rearranged product **2** was formed in excellent yield after 1.5 h only by exchanging of the solvent to [Hmim] BF_4_ as an acidic ionic liquid (Table 1, entry 4). The best result was achieved with entering of only 2 drops of DMSO to the reaction medium which accelerate, to some extent, this rearrangement so that **2** was produced in 90% yield with concomitant formation of **3** in 6% yield after only 1 h (Table 1, entry 5). This reductive rearrangement was unsuccessful again using [EMIM]Br and [HexMIM]I as other ionic liquids or under solvent-free condition. In these cases, the starting material **1** completely remained after 5 h (Table 1, entries 6–8). On the other hand, decreasing of the reaction temperature caused a decrease in the yield of **2** so that **1** remained intact completely at room temperature after 6 h (Table 1, entries 9–13). However, the result did not improve with increasing of the reaction temperature (Table 1, entry 14). Also, decreasing or even increasing of the molar ratio of Zn caused a decrease in the yield of the desired rearranged product **2** (Table 1, entries 15 and 16). In addition, this rearrangement completely failed to run in the absence of zinc powder (thermally condition) indicating its reductive nature (Table 1, entry 17). Finally, with the above study, the conditions mentioned in the entry 5 of Table 1 were selected as optimized conditions for this rearrangement and applied for other 1-(*N*-prop-2′-enylamino)anthraquinones for their reductive aza-Claisen rearrangement to 1-amino-2-(prop-2′-enyl)anthraquinones. The results are shown in Table 2. All of these 1-(*N*-prop-2′-enylamino)anthraquinones were synthesized from the reaction of the corresponding 1-aminoanthraquinones with allyl bromide in the presence of sodium hydroxide in DMF as solvent at 75 °C with reaction times from 2.5 h up to 48 h.

As shown in Table 2, different 1-(*N*-prop-2′-enylamino)anthraquinones are converted to 1-amino-2-(prop-2′-enyl)anthraquinones via reductive aza-Claisen rearrangement in good to excellent yields using the present method. It is important to note that no anthracenone formation and also double bond isomerization or reduction was observed in this method. Also, in the case of 1,5-bis(*N*- prop-2′-enylamino)anthraquinone **6** with two *N*-allyl groups, the selective formation of mono- or double-rearranged product as main product can be easily controlled only with controlling of the molar ratio of zinc powder and reaction times (Table 2, entries 3 and 4). In addition, we found that it is possible to operate this rearrangement on **13** as a tertiary amine so that **14** was produced in good yield after 1.5 h (Table 2, entry 7). Also, extending of the present procedure on 1-(*N*-prop-2′-ynylamino)anthraquinones caused efficient production of 1,2,3,4-tetrahydronaphtho[2,3-*h*]quinoline-7,12-diones via a reductive aza-Claisen rearrangement followed by cyclization in a tandem manner. Of course, the reaction temperature was reduced to 140 °C for this type of substrates (Table 2, entries 9–11). These substrates were synthesized from the reaction of the corresponding 1-aminoanthraquinones with propargyl bromide in the presence of sodium hydroxide in DMF as solvent at 75 °C in 24 h. The aromatic aza-Claisen rearrangement of arylpropargylammonium salts to 2-propargylanilines in the first step and cyclization of the rearranged products with aluminum chloride to create a new five-membered ring affording indoles in the second step has been reported [24]. However, in our work, a new six-membered heterocyclic ring is created on anthraquinone system via spontaneous cyclization of the rearranged product affording 1,2,3,4-tetrahydronaphtho[2,3-*h*]quinoline-7,12-diones in a one-pot way. However, deallylated or depropargylated products are also created in low yields in these rearrangements.

**Figure 2:**
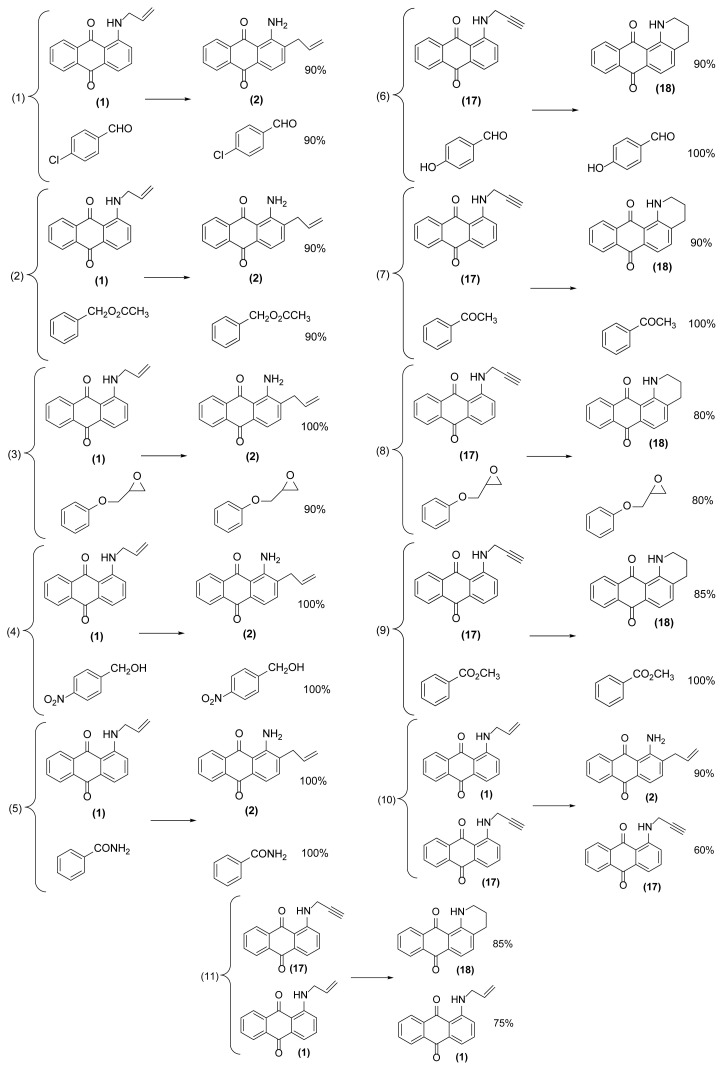
Various selectivities in the reductive aza-Claisen rearrangement of 1-(*N*-prop-2′-enylamino)anthraquinones or 1-(*N*-prop-2′-ynylamino)anthraquinones using zinc powder in [Hmim] BF_4_/DMSO at 160 °C after 1 h or 140 °C after 4.5 h, respectively.

In order to better understand the efficiency and other aspects of selectivity of this method, different competitive reactions were designed and operated (Figure 2). In each of these reactions, Zn (2 equiv.) was treated with a mixture containing 1-(*N*-prop-2′-enylamino)anthraquinone **1** or 1-(*N*-prop-2′-ynylamino)anthraquinone **17** and another compound having a specified functional group (1:1) in [Hmim] BF_4_/DMSO (2 drops) at 160 °C for 1 h or 140 °C for 4.5 h, respectively.

**Figure 3:**
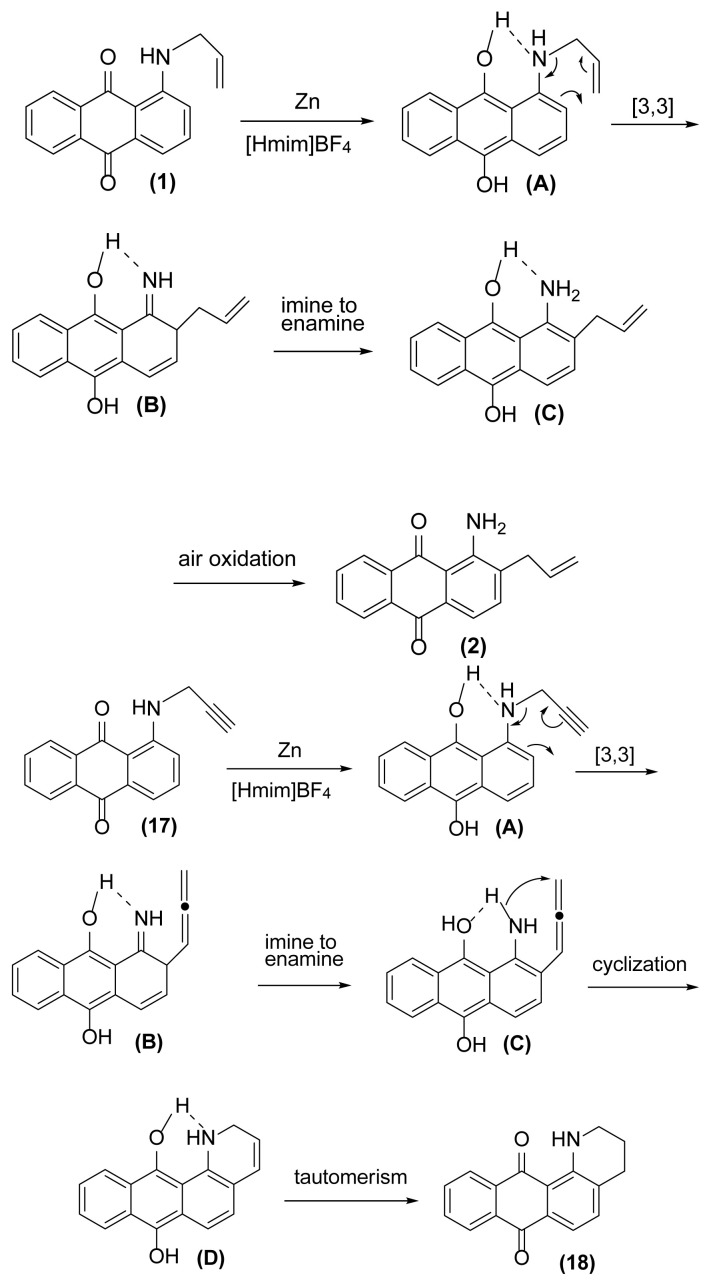
The suggested mechanism of the reductive aza-Claisen rearrangement of 1-(*N*-prop-2′-enylamino)anthraquinone and 1-(*N*-prop-2′-ynylamino)anthraquinone systems using zinc powder in [Hmim] BF_4_. N-propargyl rearrangement

As shown in Figure 2, this reductive rearrangement is executable on *N*-allyl (or *N*-propargyl)aminoanthraquinones in the presence of aldehyde, ketone, alcohol, ester, epoxide, carboxylic amide, phenolic, and nitro functional groups with excellent chemoselectivity. Also, surprisingly, it was found that the selectivity pathway for a binary mixture containing **1** and **17** is controlled by adjustment of the reaction temperature so that at 160 °C the rearranged product **2** and at 140 °C the rearranged product **18** was selectively formed as main product after 1 h and 4.5 h respectively (Figure 2, entries 10 and 11). As mentioned in Table 1, this reaction was unsuccessful in the absence of [Hmim] BF_4_ or Zn powder (thermally conditions). In these cases, the desired rearranged product was produced in only 0%–30% yield. Thus, in the mechanism of this reaction, it is proposed that anthraquinone core is reduced by Zn powder in [Hmim] BF_4_ as an acidic ionic liquid to its electron-rich hydroquinone type **A** which is more ready for this rearrangement (Figure 3).

Operation of this [3,3] sigmatropic reaction followed by enamine formation causes the creation of **C** which undergoes air oxidation to produce **2** as final rearranged product. It seems that the acidic nature of [Hmim] BF_4_ facilitates this rearrangement. In the case of *N*-propargylaminoanthraquinones; the same reduction and [3,3] sigmatropic reaction followed by enamine formation are operated affording allenic intermediate **C**. However, in continuation, this intermediate is entered in cyclization reaction followed by enol-keto tautomerism affording the final product **18** needless to air oxidation. In accordance with these mechanisms, when the present procedure was applied on **1** and also **17** separately under N_2_ atmosphere, no product formation was observed in the case of *N*-allyl (i.e. **1**) after 5 h while **18** was produced in 80% yield after the same reaction time indicating that air oxidation is necessary to produce rearranged product from *N*-allylaminoanthraquinones but not from *N*-propargylaminoanthraquinones.

## 4. Conclusion

In conclusion, the present study introduces, for the first time, reductive aza-Claisen rearrangement of 1-(*N*-allylamino)anthraquinones to 1-amino-2-(prop-2′-enyl)anthraquinones using zinc powder in [Hmim] BF_4_ as an ionic liquid in good to excellent yields. No anthracenone formation and also double bond isomerization or reduction was observed in this method.

Also, 1-(*N*-propargylamino)anthraquinones is converted to 1,2,3,4-tetrahydronaphtho[2,3-*h*]quinoline-7,12-diones containing a newly synthesized six-membered heterocyclic ring on anthraquinone core under these conditions via this [3,3] sigmatropic rearrangement followed by cyclization in a tandem manner. Surprisingly, with adjustment of the reaction temperature, it is possible to operate reductive aza-Claisen rearrangement of *N*-allylaminoanthraquinones in the presence of *N*-propargylaminoanthraquinones or vice versa with good selectivity. Moreover, some different other functional groups well tolerate this rearrangement so that it is executable with excellent chemoselectivity. In addition, operation in ionic liquid as environment-friendly solvent can be considered another advantage of the present method.

## Figures and Tables

**Table 1:** Reductive aza-Claisen rearrangement of 1-(*N*-prop-2′-enylamino)anthraquinone **1** to 1-amino-2-(prop-2′-enyl)anthraquinone **2** using zinc powder in different conditions.

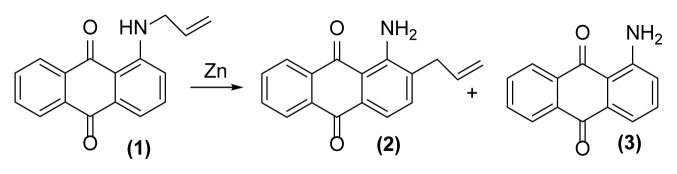						
Entry	Molar ratio of Zn	Solvent	Temp. (°C)	Time (h)	Yield (%) of **2**	Yield (%) of **3**^a^
1	2	AcOH	Reflux	4.5	20	80
2	2	DMF	Reflux	5	25	75
3	2	DMSO	160	5	30	70
4	2	[Hmim] BF_4_	160	1.5	90	10
5	2	[Hmim] BF_4_^b^	160	1	90	6
6	2	[EMIM]Br^c^	160	5	0	0
7	2	[HexMIM]I^d^	160	5	0	0
8	2	Solvent free	160	5	0	0
9	2	[Hmim] BF_4_	140	4.5	65	10
10	2	[Hmim] BF_4_	120	4.5	45	25
11	2	[Hmim] BF_4_	100	5	35	25
12	2	[Hmim] BF_4_	70	8	30	20
13	2	[Hmim] BF_4_	rt	6	0	0
14	2	[Hmim] BF_4_	180	1.5	85	15
15	1	[Hmim] BF_4_	160	1.5	50	20
16	4	[Hmim] BF_4_	160	1.5	80	20
17	0	[Hmim] BF_4_	160	6	0	0

a **3** is deallylated product (1-aminoanthraquinone).

b In this case, DMSO (2 drops) was added to the reaction mixture.

c 1-Ethyl-3-methylimidazolium bromide.

d 1-Hexyl-3-methylimidazolium iodide.

**Table 2:** Reductive aza-Claisen rearrangement on anthraquinone structures using Zn powder (2 equiv.) in [Hmim] BF_4_ at 160 °C.^a^

Entry no.	Substrate	Product	Time (h)	Yield (%)^b^
1	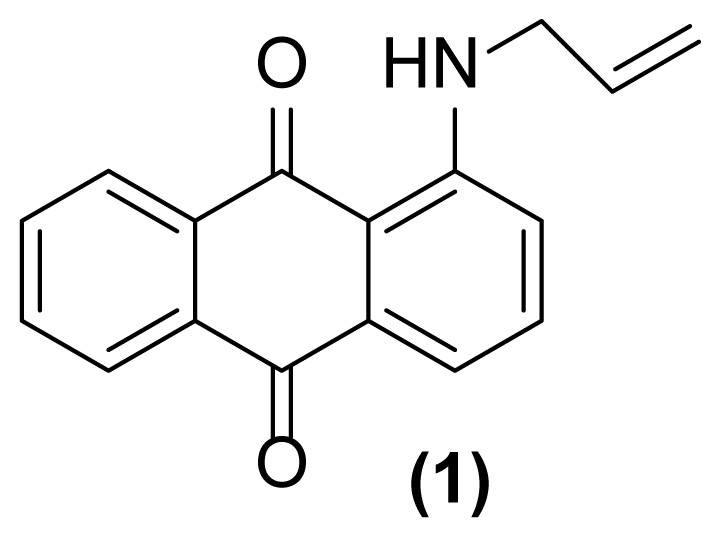	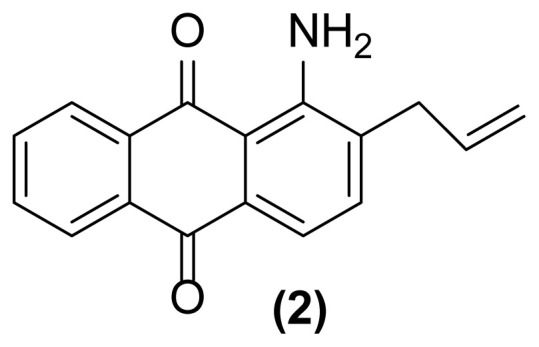 Deallylated	1	90 6
2	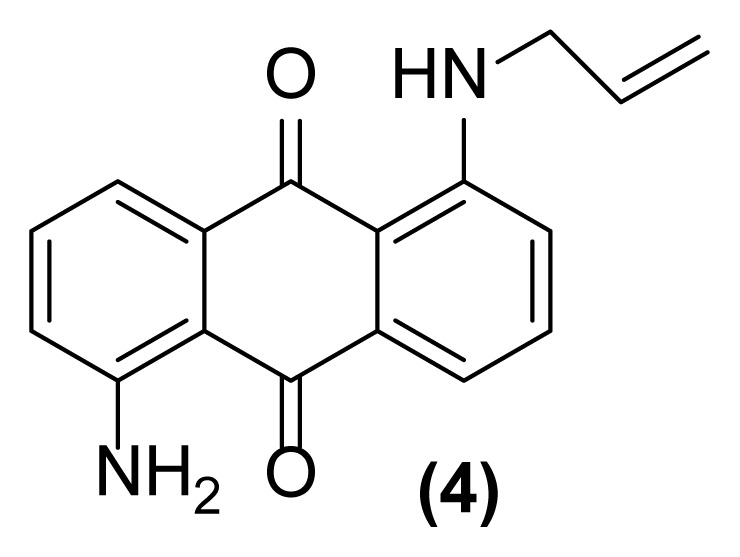	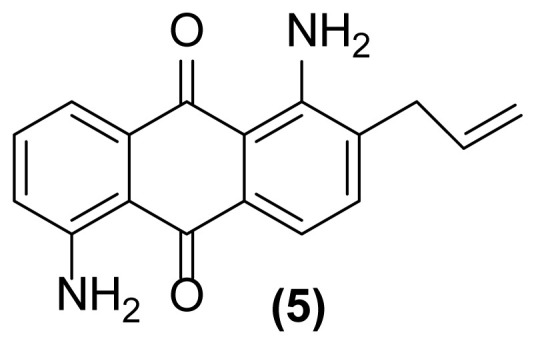 Deallylated	4.5	82 15
3	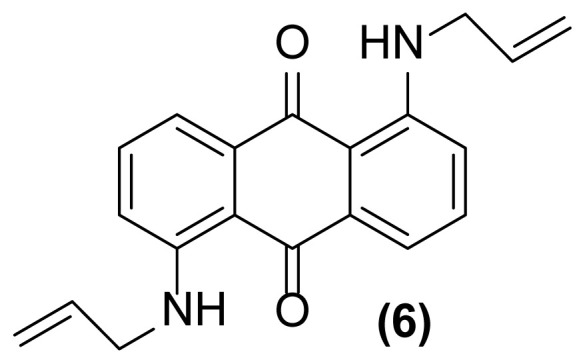	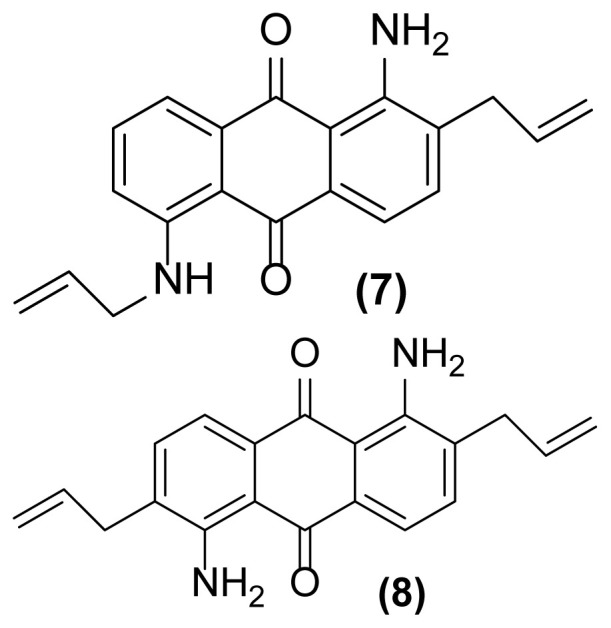 Doubly deallylated	4.5	53 35 7
4^c^	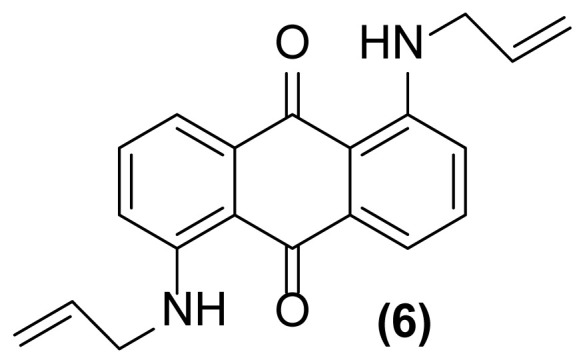	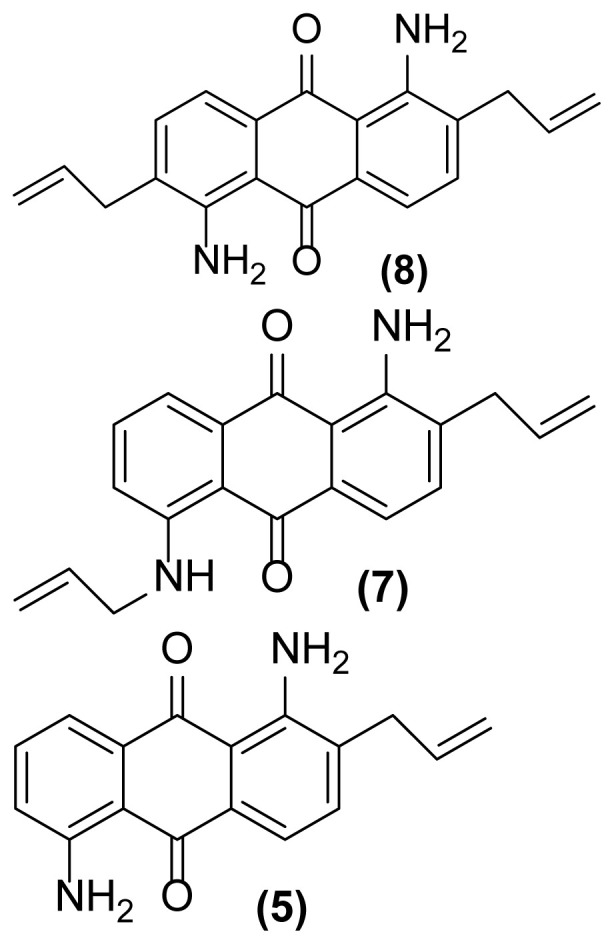 Doubly deallylated	10	64 14 10 5
5	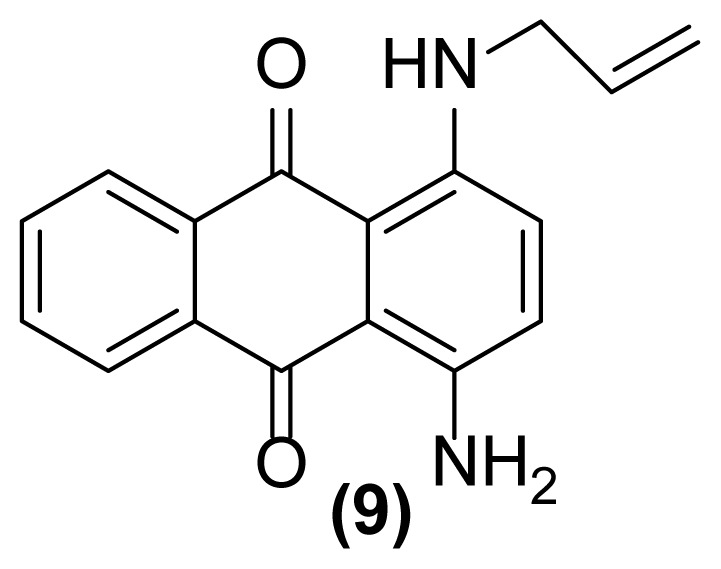	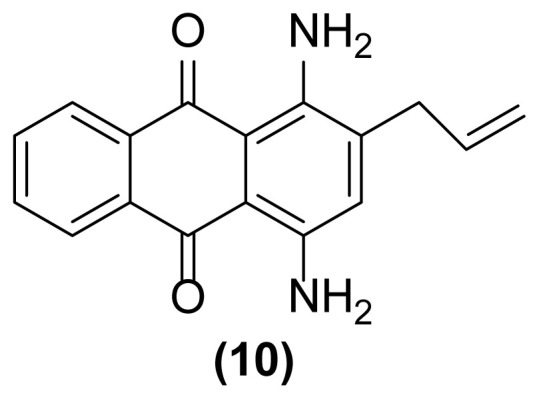 Deallylated	4.5	67 27
6	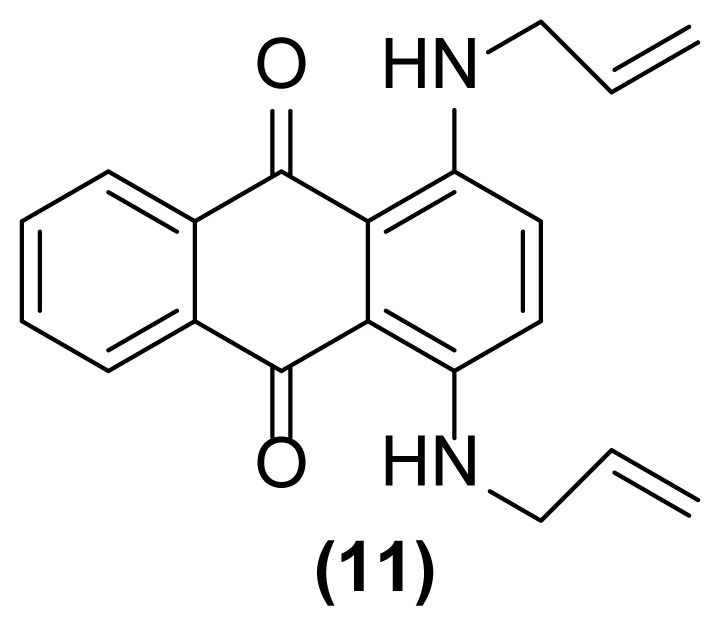	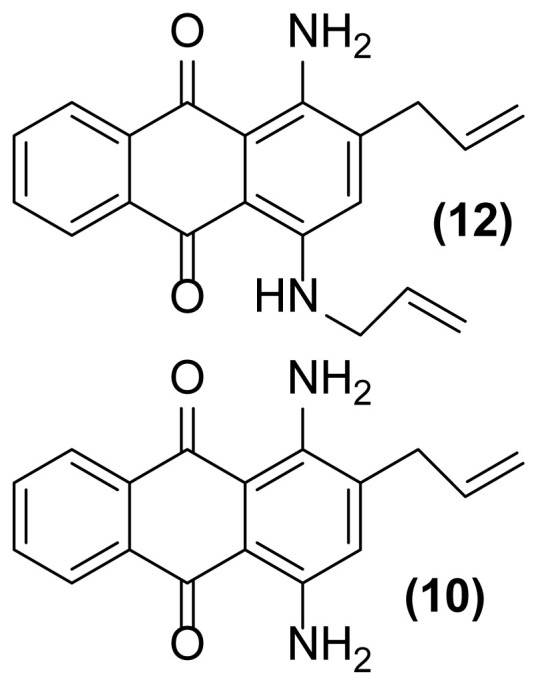 Doubly deallylated	6	61 15 20
7	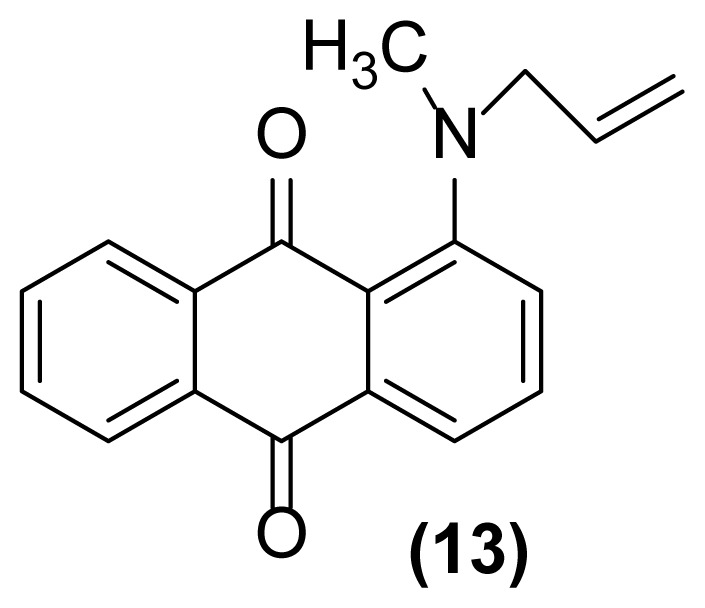	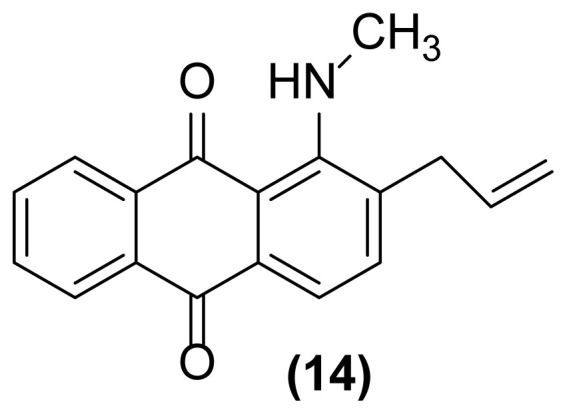 Deallylated	1.5	65 30
8	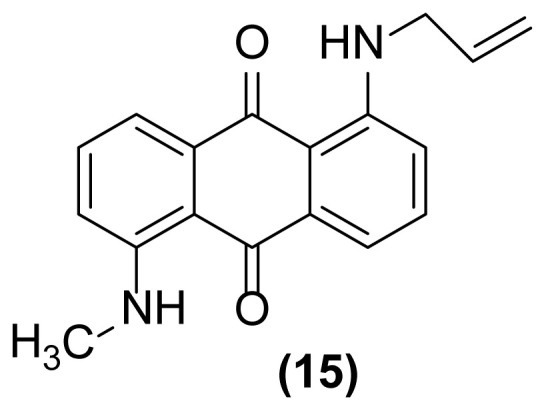	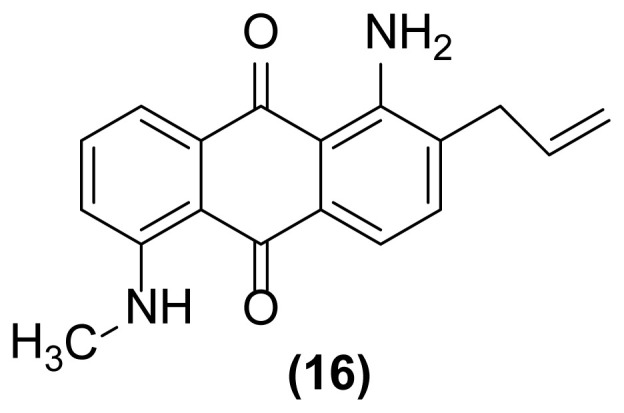 Deallylated	2.5	76 15
9^d^	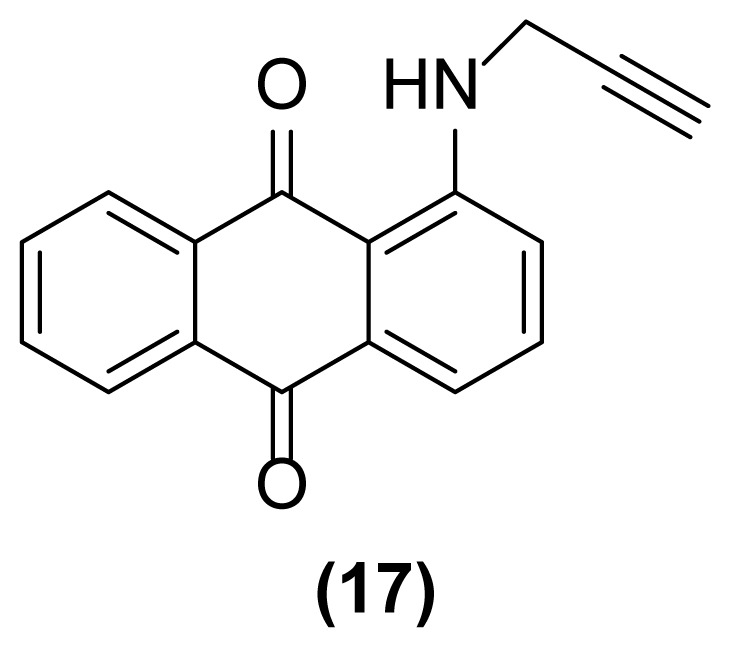	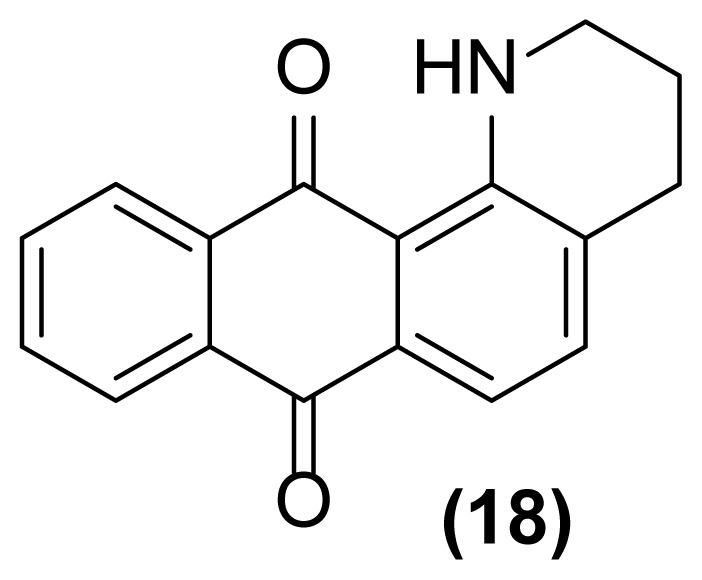 Depropargylated	5.5	91 5
10^d^	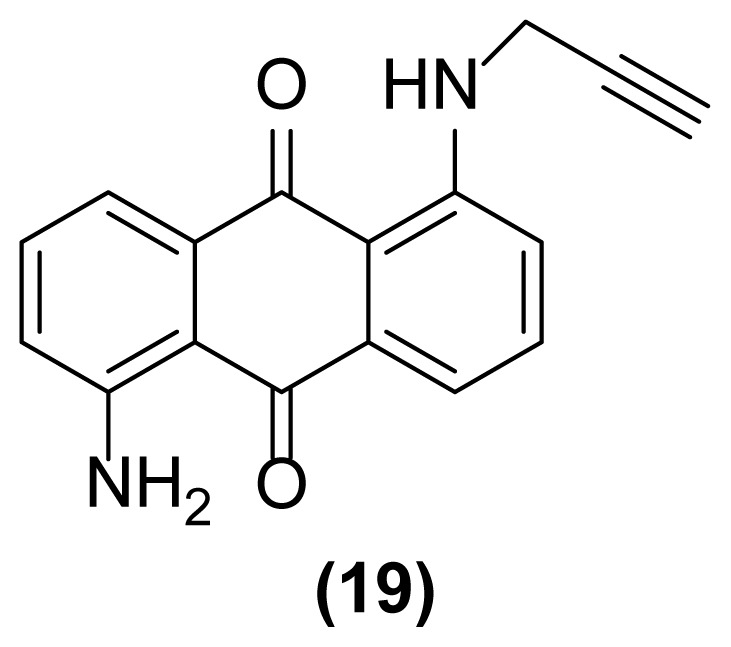	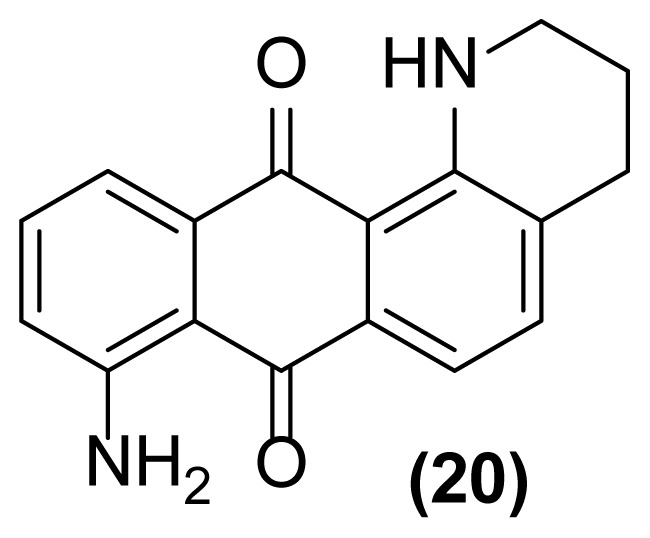 Depropargylated	5.5	77 5
11^d^	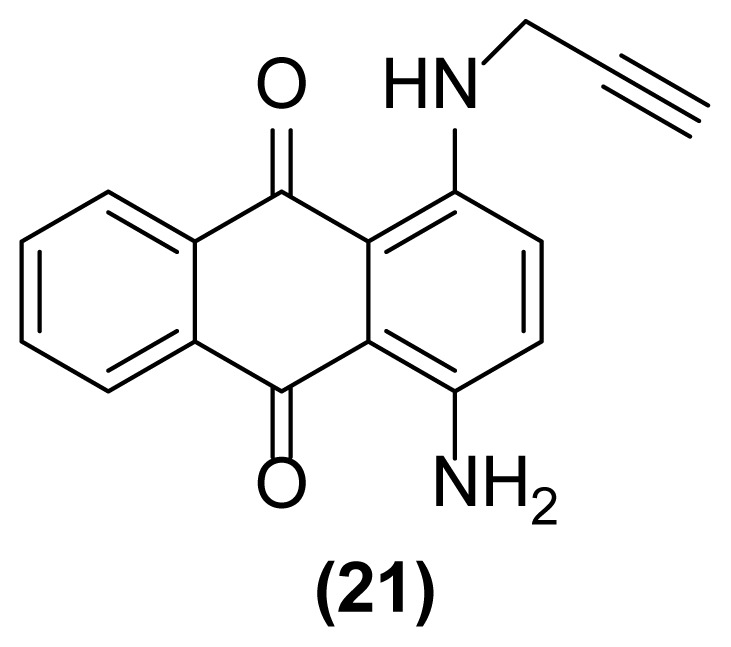	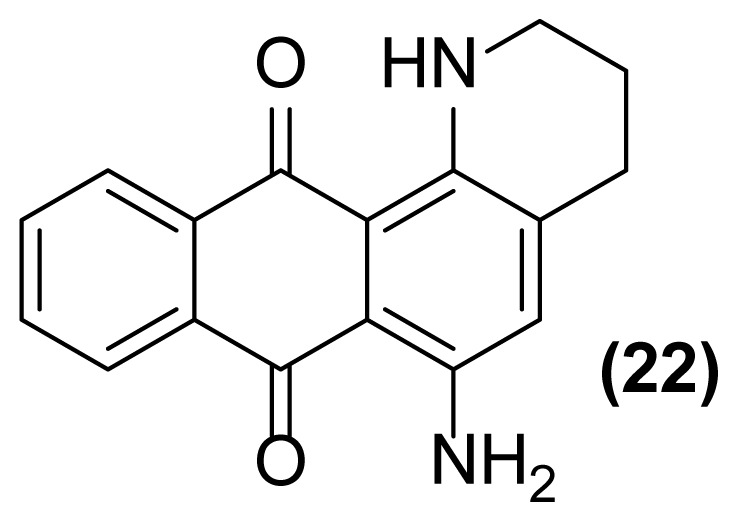 Depropargylated	5.5	73 6

a DMSO (2 drops) was added to the reaction medium in this rearrangement.

b Isolated yields.

c The molar ratio of substrate to Zn powder was adjusted to 1:3 in this case.

d This rearrangement was carried out at 140 °C.

## References

[1] Dauzonne D Fouris S General syntheses of novel anthracene-9,10-dione derivatives: the 2-(1-aryl-2-nitroethyl)-1,4-dihydroxyanthracene-9,10-diones, 2-(1-arylethenyl)-1,4-dihydroxyanthracene-9,10-diones and 2-(1-arylethyl)-1,4-dihydroxyanthracene-9,10-diones Tetrahedron 1993 49 39 8865 8876 10.1016/S0040-4020(01)81906-2

[2] Ghosh A Jose DA Kaushik R Anthraquinones as versatile colorimetric reagent for anions Sensors and Actuators B: Chemical 2016 229 545 560 10.1016/j.snb.2016.01.140

[3] Langdon-Jones EE Pope SJA The coordination chemistry of substituted anthraquinones: Developments and applications Coordination Chemistry Reviews 2014 269 32 53 10.1016/j.ccr.2014.02.003

[4] Karhunen P Brunow G Studies on the formation of benzanthrones in alkaline anthraquinone pulping Acta Chemica Scandinavica 1991 45 945 948 10.3891/acta.chem.scand.45-0945

[5] Yen G-C Duh P-D Chuang D-Y Antioxidant activity of anthraquinones and anthrone Food Chemistry 2000 70 4 437 441 10.1016/S0308-8146(00)00108-4

[6] Wuthi-udomlert M Kupittayanant P Gritsanapan W In vitro evaluation of antifungal activity of anthraquinone derivatives of Senna alata Journal of Health Research 2010 24 3 117 122

[7] Augustin N Nuthakki VK Abdullaha M Hassan QP Gandhi SG Discovery of Helminthosporin, an Anthraquinone Isolated from Rumex abyssinicus Jacq as a Dual Cholinesterase Inhibitor ACS Omega 2020 5 1616 1624 10.1021/acsomega.9b03693 32010836 PMC6990627

[8] Davis R Agnew P Shapiro E Antiarthritic activity of anthraquinones found in aloe for podiatric medicine Journal of the American Podiatric Medical Association 1986 76 2 61 66 10.7547/87507315-76-2-61 3941379

[9] Hou Y Cao S Brodie PJ Callmander MW Ratovoson F Antiproliferative and antimalarial anthraquinones of Scutia myrtina from the Madagascar forest Bioorganic & Medicinal Chemistry 2009 17 7 2871 2876 10.1016/j.bmc.2009.02.022 19282186 PMC2728447

[10] Prateeksha Yusuf MA Singh BN Sudheer S Kharwar RN Chrysophanol: A Natural Anthraquinone with Multifaceted Biotherapeutic Potential Biomolecules 2019 9 2 68 10.3390/biom9020068 30781696 PMC6406798

[11] Wang J Qin X Chen Z Ju Z He W Two new anthraquinones with antiviral activities from the barks of *Morinda citrifolia* (Noni) Phytochemistry Letters 2016 15 13 15 10.1016/j.phytol.2015.11.006

[12] Huang Q Lu G Shen H-M Chung MCM Ong CN Anti-cancer properties of anthraquinones from rhubarb Medicinal Research Reviews 2007 27 5 609 630 10.1002/med.20094 17022020

[13] Abu N Akhtar MN Ho WY Yeap SK Alitheen NB 3-Bromo-1-Hydroxy-9,10-Anthraquinone (BHAQ) Inhibits Growth and Migration of the Human Breast Cancer Cell Lines MCF-7 and MDA-MB231 Molecules 2013 18 9 10367 10377 10.3390/molecules180910367 23985955 PMC6269781

[14] Zhang Z Wu X-H Sun F-Q Shan F Chen J-C Synthesis, characterization of ruthenium(II) complex of 1,3,8-trihydroxy-6-methyl-anthraquinone (emodin) and its binding behavior with *c-myc* G-quadruplex Inorganica Chimica Acta 2014 418 23 29 10.1016/j.ica.2014.04.014

[15] Choi HK Ryu H Son A Seo B Hwang S-G The novel anthraquinone derivative IMP1338 induces death of human cancer cells by p53-independent S and G2/M cell cycle arrest Biomedicine & Pharmacotherapy 2016 79 308 314 10.1016/j.biopha.2016.02.034 27044842

[16] Tian W Wang C Li D Hou H Novel anthraquinone compounds as anticancer agents and their potential mechanism Future Medicinal Chemistry 2020 12 7 627 644 10.4155/fmc-2019-0322 32175770

[17] van Gorkom BAP de Veries EGE Karrenbeld A Kleibeuker JH Review article: anthranoid laxatives and their potential carcinogenic effects Alimentary Pharmacology & Therapeutics 1999 13 4 443 452 10.1046/j.1365-2036.1999.00468.x 10215727

[18] Park M-Y Kwon H-J Sung M-K Evaluation of Aloin and Aloe-Emodin as Anti-Inflammatory Agents in Aloe by Using Murine Macrophages Bioscience, Biotechnology & Biochemistry 2009 73 4 828 832 10.1271/bbb.80714 19352036

[19] Martin Castro AM Claisen rearrangement over the past nine decades Chemical Review 2004 104 2939 3002 10.1021/cr020703u 15186185

[20] (a) Murty KVSN Pal R Datta K Mal D Glucose Promoted Claisen Rearrangement of 1-Allyloxy Anthraquinones Synthetic Communication 1994 24 9 1287 1292 10.1080/00397919408011730

[21] (a) Cambie RC Milbank JBJ Rutledge PS Reductive Claisen rearrangements of allyloxyanthraquinones. A review Organic Preparations and Procedures International 1997 29 4 365 407 10.1080/00304949709355216

[22] Majumdar KC Bhattacharyya T Chattopadhyay B Sinha B Recent advances in the aza-Claisen rearrangement Synthesis 2009 13 2117 2142 10.1055/s-0029-1217389

[23] (a) Dzieszkowski K Baranska I Rafinski Z Construction of Dihydropyrido[2,3-d]pyrimidine Scaffolds via Aza-Claisen Rearrangement Catalyzed by N-Heterocyclic Carbenes The Journal of Organic Chemistry 2020 85 6645 6662 10.1021/acs.joc.0c00657 32312044 PMC7590975

[24] Han L Li S-J Zhang X-T Tian S-K Aromatic Aza-Claisen Rearrangement of Arylpropargylammonium Salts Generated in Situ from Arynes and Tertiary Propargylamines Chemistry-A European Journal 2021 27 3091 3097 10.1002/chem.202004356 33205537

[25] Jain S Pandey N Kishore D Lewis acid catalyzed amino-Claisen rearrangement: A facile one-pot synthesis of 2-allylarylamines from *N*-allylarylamines Indian Journal of Chemistry 2007 46B 529 531

[26] Sharghi H Aghapour G Claisen Rearrangement of Allyloxyanthraquinones with Silver/Potassium Iodide in Acetic Acid as a New and Efficient Reagent The Journal of Organic Chemistry 2000 65 9 2813 2815 10.1021/jo991674z 10808462

[27] Nadali S Aghapour G Rafieepour Z Efficient and selective iron-mediated reductive Claisen rearrangement of propargyloxyanthraquinones to anthrafurandiones in ionic liquids Canadian Journal of Chemistry 2017 95 10 1045 1051 10.1139/cjc-2017-0328

[28] Nadali S Khoshroo A Aghapour G Efficient reductive Claisen rearrangement of prop-2′-enyloxyanthraquinones and 2′-chloroprop-2′-enyloxyanthraquinones with iron powder in ionic liquids Turkish Journal of Chemistry 2018 42 3 883 895 10.3906/kim-1711-49

[29] Zhu H-P Yang F Tang J He M-Y Brønsted acidic ionic liquid 1-methylimidazolium tetrafluoroborate: a green catalyst and recyclable medium for esterification Green Chemistry 2003 5 38 39 10.1039/b209248b

